# The Prophylaxis of Influenza

**Published:** 1918-11

**Authors:** 


**The Prophylaxis of Influenza.** *La Presse Médicale*, October 17, 1918.

The "Académie de Médecine" adopted the following conclusions of Mr. Bezançon's report on the prophylactic measures against influenza.

Influenza must be considered as an eminently infective specific disease; the incubation period is extremely short. Some facts seem to demonstrate that a first attack of the disease confers a certain immunity.

The secondary infections arising as complications of influenza are also most contagious.

The spreading of the disease is essentially intrahuman; it is favored by crowds and insufficient ventilation.

Prophylactic measures imply :

1) Avoiding all contact with the patients and making careful antiseptic washings of mouth and rhino-pharynx. Infection is most likely to occur in crowded places, especially in dark and badly ventilated rooms, theaters, cinemas.

Complete and repeated disinfections of the metropolitan, tramways and buses are most advisable.

2) Patients should be isolated from each other and from visitors. This measure, if strictly applied, proves most effective.

Simple cases should be separated from complicated ones and this should be done as well in military and civil hospitals as in private houses.

Convalescents should be directed as soon as possible to special hospitals.

The entrance of the influenza wards should be strictly forbidden to visitors, except for very serious reasons; and a special staff of attendants should be designated for those wards.

Masks similar to those adopted by the Americans in such cases are highly recommended for all persons attending to influenza cases, and for the patients themselves at the beginning of convalescence.

The Commission approves of all prescriptions ordered by the Sanitary Service about the prophylaxis of influenza, among both soldiers and civilians, and insists quite particularly upon the necessity of isolating most strictly the influenza cases from other patients, especially from wounded or gassed soldiers.

It is desirable that influenza cases should be spared long journeys and should be cared for in the nearest possible hospital. If long journeys must be undertaken, special well-heated and repeatedly disinfected sanitary trains are far more advisable than automobile ambulances for such cases.

